# Better late than never!—a long-awaited necessary turn in the prenatal management of early 2nd trimester LUTO

**DOI:** 10.1007/s00467-026-07188-8

**Published:** 2026-02-07

**Authors:** Thomas Kohl

**Affiliations:** German Center for Fetal Surgery & Minimally-Invasive Therapy (DZFT), University of Heidelberg, Campus Mannheim, Theodor-Kutzer-Ufer 1-3, 68167 Mannheim, Germany

Dear Editor,

As the first proponent of a systematic management algorithm for fetuses with early second-trimester LUTO that departed from all previous management recommendations for vesico-amniotic shunt insertions (VAS) [1], I was positively surprised that the authors of “Early diagnosis and intervention in congenital lower urinary tract obstruction: time to revise our approach?”, published in Pediatric Nephrology in November 2025 [2], consider—obviously with a grain of salt—the previously unthinkable: opening up for early VAS for those fetuses that are detected in the early second trimester of pregnancy.

Despite being familiar with the mostly fatal outcomes of early severe LUTO, the long-standing availability of experimental pathophysiological studies that favor early treatment, and the glaring example of the fatal consequences of a failed randomized trial, the authors clamor for “large-scale, multidisciplinary prospective studies” and novel biomarkers for prognostication.

What at first glance seems cautious and appropriate risks once again to result in only mediocre results from relying on still unsuitable shunt designs, relying on variably skilled interventionists, and accommodating to procedures performed in suboptimal hygienic and interventional settings [3]. The promise of novel “biomarkers” that may obviate the time-robbing wait for serial urinary analysis will not unburden today’s clinicians from their responsibility to already offer early VAS from the encouraging data that already has been provided by myself and my other German colleagues. And, concerning ethics, I strongly suggest to *not* withhold treatment from female LUTO fetuses and those with urethral atresia.
